# In vitro fertilization and the ethics of frozen embryos

**DOI:** 10.1038/s44319-024-00161-2

**Published:** 2024-05-20

**Authors:** Shina Caroline Lynn Kamerlin

**Affiliations:** Georgia Institute of Technology, Atlanta, GA USA

**Keywords:** Economics, Law & Politics, Stem Cells & Regenerative Medicine

## Abstract

The Alabama Supreme Court decision to consider embryos as children sheds light on a growing problem for IVF clinics: what to do with the increasing number of frozen surplus embryos?

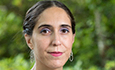

Given the sensitive nature of the topic I am writing about, I want to clarify two important points first. I fully support the use of assisted reproductive technologies (ART) and would personally not be a parent without them; and the goal of this column is not to provide a value judgment on what should be done with leftover/abandoned in vitro fertilization (IVF) embryos, but rather to shine light on the problem of ever larger numbers of embryos that are being created.

On February 16th, the Alabama Supreme Court ruled that embryos generated through IVF should be considered to have the same rights as children, a surprising ruling with major ethical and legal consequences and potentially disastrous consequences for fertility treatment in the state. Unsurprisingly, this ruling met with immediate criticism, including from the American College of Obstetricians and Gynecologists who expressed their strong opposition to conferring “personhood” to fertilized embryos, eggs or fetuses. One of their concerns is the fear that it might open the doors to potential criminal liability for pregnant women managing their own health.

The discussion surrounding legal personhood for frozen embryos is not new, however. As soon as *Roe v. Wade* was overturned by the US Supreme Court last year, my own thought was “what does this mean for IVF embryos?” In fact, this issue has been discussed more thoroughly by legal experts (see e.g., (Letterie and Fox, 2023)). On an international level, discussions of embryonic personhood predate the Alabama Supreme Court decision (Dickens, 2016), and at least some sort of “regard” for human embryos has been incorporated into global legal considerations (Millibank, 2017; EC Working Group on human embryos and research, 1995). The idea that embryos are not just any other human tissue and should not be treated as such is a noble principle. But, when enshrined into law, it has major consequences in light of the growing number of frozen embryos resulting from IVF treatment, because it limits if and how these embryos can be disposed of.

In practical terms, undergoing IVF is both an unpleasant experience in terms of medication and side effects, and it can be an extremely costly procedure. In Europe, a number of countries offer at least a few cycles of IVF on public health systems, but further attempts would have to be paid for out of pocket at private clinics. Although there is a move towards at least some insurance coverage of fertility treatment in the USA, this is invariably limited. These factors combined make it easier and more economically favorable for patients and clinics to attempt to create as many embryos as possible at once to maximize the chance of success. Even so, the nature of IVF is such that typically more embryos are created than used. These so-called surplus embryos can typically be frozen and stored, and an ever-increasing number survive the thawing process as cryopreservation techniques are being improved. Embryos can thus, in principle, be stored indefinitely either for potential later use or as it seems to be ethically or legally defensible.

From an individual perspective, this might not seem to be an issue: an embryo is small, and therefore storing it indefinitely may appear trivial. However, while individual embryos are small, the liquid nitrogen tanks in which they are stored are not, and the space and energy footprint needed becomes a serious problem, especially given that not insignificant numbers of surplus embryos are considered “abandoned” (unclaimed) with no obvious endpoint to how long they need to be stored. As some examples of the magnitude of the problem, it has been estimated that, in 2016, between 400,000 and 1.4 million frozen embryos were stored in the USA. According to the Spanish Fertility Society, as of 2023, there were more than 600,000 cryopreserved embryos in Spain, of which ~60,000 were estimated to be “abandoned”. The UK apparently freezes around 100,000 embryos per year, with IVF providers raising the alarm that they are running out of room. These are formidable numbers that keep growing every year with currently no clear ethical or legal means to reach a practical solution.

In practice, there are many options for surplus frozen embryos: they can be donated to aspiring parents (embryo adoption); it is possible to perform a “compassionate transfer” at a time in the woman’s cycle where pregnancy is least likely; they can be donated to science; they can be destroyed; or they can be stored, typically against a recurring storage fee. While these are all reasonable alternatives, a major challenge is that a large number of embryos are simply abandoned, which poses major ethical and logistical headaches for the fertility clinics storing them (Cattapan and Baylis, 2015). They are trapped in a Catch-22 situation: discarding unwanted embryos may have them facing the wrath of either parents who change their minds and want to reclaim their embryos, or the law, as embryo destruction is not a legal option in many countries; on the other hand, storing them indefinitely is not really a scalable option either.

These challenges are amplified by broader differences in national and international regulations of fertility treatment, treatment protocols, and, most importantly, how long embryos should or could be stored for. In terms of access to fertility treatment, 38 out of 43 European countries had dedicated laws on reproductive technologies, according to data from Fertility Europe as of December 2021. However, in terms of the specifics—for instance who can receive treatment and what kind of treatment they can receive—these laws vary widely between countries. In comparison, assisted reproductive technology is heavily regulated at the point of access in the USA, both at the state and federal level, with a national body, the American Society for Reproductive Medicine, providing oversight.

Most important to the current discussion, however, is that there are also vastly different regulations regarding how long frozen embryos can be stored. By way of examples, frozen eggs, sperm and embryos, can only be stored for a maximum of 55 years in the UK. In Sweden, frozen embryos can be stored for a maximum of 10 years (increased from 5 in 2019). In Australia, embryos can be stored for a maximum of 5 years. In the USA, however, both eggs and embryos can be frozen indefinitely. While cost can be a barrier for parents, leading some families to opt for destroying embryos instead of paying for continuous storage, the ability to store frozen embryos indefinitely contributes to the problem of embryo abandonment, and the associated logistical and ethical dilemmas.

How to solve this problem I cannot answer, given the myriad of associated ethical issues, but it’s becoming clear that as the growing number of frozen embryos grows is a problem we cannot keep side-stepping.

### Supplementary information

Peer Review File
