# Supplementary material for: In vitro fertilization and the ethics of frozen embryos
Source: EMBO Rep. 2024 May 20;25(7):2817–8. doi: 10.1038/s44319-024-00161-2 (PMC11239656; doi:10.1038/s44319-024-00161-2)
Supplement: Supplementary file 1 — Peer Review File [file 44319_2024_161_MOESM1_ESM.pdf]

EMBO reports

In vitro fertilization and the ethics of frozen embryos

Shina Caroline Lynn Kamerlin

Corresponding author(s): Shina Caroline Lynn Kamerlin (skamerlin3@gatech.edu)

Review Timeline:

	Submission Date:	8th May 24
	Accepted:	8th May 24

Editor: Holger Breithaupt

Transaction Report: This article type is not peer reviewed.
